# Effects of *Allium roseum* L. extracts on the proliferation and the differentiation of the acute myeloid leukemia cell line U937

**DOI:** 10.1002/fsn3.2965

**Published:** 2022-07-27

**Authors:** Abdelkarim Ben Arfa, Mondher Boulaaba, Faten Merhi, Brigitte Bauvois, Arnault Ingrid, Jacques Auger, Mohamed Neffati, Hanen Najjaa

**Affiliations:** ^1^ Laboratoire des Ecosystèmes Pastoraux et de Valorization des Plantes Spontanées Institut des Régions Arides Université de Gabès Médenine Tunisie; ^2^ Laboratoire des Plantes Aromatiques et Médicinales, Centre de Biotechnologie Technopark de Borj‐Cédria (CBBC) Hammam‐Lif Tunisie; ^3^ INSERM U872, Université Pierre et Marie Curie, Université Paris Descartes Centre de Recherche des Cordeliers Paris France; ^4^ IRBI, UMR CNRS 6035 Université François Rabelais Tours France

**Keywords:** *Allium roseum*, cytotoxicity, differentiation, leukemia, matrix metalloproteinase‐9, proliferation

## Abstract

Epidemiologic studies keep up the proposition that *Allium* vegetables can lower the risk of cancers. Acute myeloid leukemia (AML) cells exhibit high proliferative potency and have a reduced capacity of undergoing apoptosis and maturation. The beneficial effects of *Allium* seem related to the organosulfur products generated upon processing of these species. For this purpose, the aim of this study was to test *Allium roseum* fresh (FAE), crude (CAE) and dried (DAE) aqueous extracts for activity against the human acute leukemia cell line (U937). As assessed by flow cytometry, inhibited cell proliferation was in a dose‐dependent manner. Firstly, study showed that cell growth was inhibited with 20 mg/mL using FAE and CAE (60% and 73% respectively). Secondly, our experiments clearly indicate that all *A. roseum* extracts do not induce cell apoptosis. This was confirmed by the soft binding of Annexin V to phosphatidylserine. Finally, the high expression of macrophage's marker CD11 associated with adequate morphological changes proves clearly the differentiation aspect produced by *A. roseum* extract. Taken together, these data suggest that *A. roseum* could be a promising candidate for the alternative medicine in the field of cancer therapy.

## Graphical abstract

Allium roseum extracts inhibited cell proliferation of human acute leukemia cell line U937 in dose‐dependent manner.

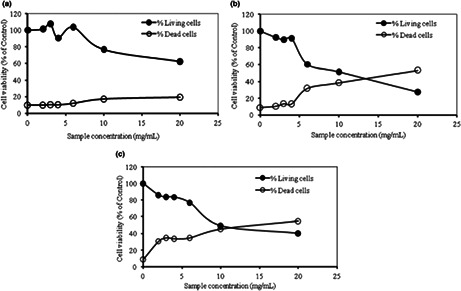

## 1 INTRODUCTION

Leukemia which is found in blood and bone marrow is one of serious type of cancer and a major cause of cancer death worldwide (Siegel et al., 2015; Hassanpour & Karami, 2018); about 5% of all cancer cases ranking sixth among various human malignant tumors (Zhang et al., 2012). Additionally, it is the neoplasm that causes about 30% of all cancer‐related deaths in children (Katrin et al., 2017). Leukemia is considered chronic or acute. Acute myeloid leukemia (AML) is an aggressive malignancy characterized by rapid growth of abnormal white blood cells. AML is primarily treated by chemotherapy, and radiotherapy is rarely applied. Although conventional chemotherapy of AML with either cytarabine or daunorubicin given as a single agent induces complete remission in around 30%–40% of patients and combination treatment with both agents induces complete remission in more than 50% of patients. In that case, only 20%–30% of patients enjoy long‐term disease‐free survival and these chemo drugs can also affect normal cells causing unpleasant side effects, such as anemia, bleeding, and infection (Hsiao et al., 2013). Hence, the development of new compounds directed against leukemia‐specific targets is however needed to increase the cure rate in AML patients exhibiting chemoresistance and poor outcomes. Natural products and plant extracts have been investigated extensively for potential anticancer activity in a number of solid and hematological malignancies, including AML, CML, Hodgkin's and non‐Hodgkin's lymphomas and lymphoid leukemias (Tawil et al., 2015). However, the potential of naturally occurring products in AML has not been fully explored yet, hence the need for identifying novel plant‐derived compounds for potential anti‐AML activity. Among the species studied, the members of the genus Allium have received a special attention. *Allium* species are widely consumed as food worldwide and impact significantly on human health for their high content of bioactive compounds that act as antioxidants, antimicrobial, antiinflammatory, and anticancer activity (Rocchetti et al., 2022).

*Allium roseum* (Amaryllidaceae Family) is a well‐known garlic and onion that is widely used in Mediterranean cuisine even in Tunisia. It is used by local consumers as a vegetable, spice, and herbal remedy. Moreover, as the authors of this paper mentioned earlier, leaves of this plant was used in folk medicine for the treatment of headaches, stomach aches, and rheumatism (Najjaa, Zria, et al., 2011). Previous works mentioned the antiproliferative (Souid et al., 2016), the antioxidant, and antimicrobial effects of *A. roseum* (Najjaa et al., 2007; Najjaa, Zouari, et al., 2011) as well as the inhibition of amyloid beta aggregation and toxicity involved in Alzheimer's disease (Boubakri et al., 2020). Recently, we reported that the dehydrated aqueous extract of *A. roseum* exhibits anti‐chronic myeloid leukemia activity through the inhibition of BCR‐ABL, PI3K/Akt, and ERK1/2 pathways and the abrogation of VEGF secretion (Souid et al., 2016) but not on the acute myeloid leukemia cells U937. Related to some of these activities, phytochemical analysis showed the presence of sulfur compounds, such as diallyl disulfide (DADS) and diallyl trisulfide (DATS) in *Allium* species which have an antiproliferative and proapoptotic effects in human epithelial cancer and neuronal cell lines (Hosono et al., 2005; Milner, 2006; Xiao et al., 2005; Xiao & Singh, 2006). Another sulfur molecule such as dipropyl thiosulfinate (Pr_2_TS) is also identified in *Allium* species (Lanzotti, 2006) and which presented an inhibition of proliferation of U937 in dose‐ and time‐dependent manner through induction of macrophage maturation and inhibition of the levels of secreted matrix metalloproteinase‐9 (MMP‐9) (Merhi et al., 2008). In addition, saponins, tannins, flavonoids, coumarins, steroids, cardiac glycosides, free quinone, and iridoids were found in *A. roseum* (Najjaa, Zria, et al., 2011).

This work herein presented the capacity of *A. roseum* to reduce the proliferation of the human acute myeloid leukemia (AML) cell line U937, apoptosis and differentiation.

## 2 MATERIALS AND METHODS

### 2.1 Sampling and sample preparation

The leaves of wild‐growing *A. roseum* were collected from the arid Southeast of Tunisia (Ben Guerdane), at the vegetative stage of the plant growing cycle (January, 2010). The Voucher specimen was deposited at the herbarium of the Range Ecology Laboratory of the “Institut des Régions Arides” of Tunisia. Three aqueous extracts from different forms of *A. roseum* subjected to different processing were prepared using the following method. 15 g of dried leaves of *A. roseum* were extracted with 100 ml of water (DAE) during 1 h at room temperature. A quantity of 30 g of fresh (FAE) and crushed (CAE) *A. roseum* leaves were extracted separately with 100 ml of distilled water for 1 h at room temperature. After centrifugation at 8000 *g*, supernatants were recovered. To prevent denaturation, extraction was achieved rapidly and extracts were immediately used or stored at 20°C until further use.

### 2.2 Cell culture and antiproliferative assay

The human acute myeloid leukemia U937 cell lines (American Tissue Cell Culture) were kindly supplied by Dr Michel Lanotte (Hôpital Saint‐Louis, Paris, France) and cultured in a 5% CO_2_ humidified atmosphere at 37°C in RPMI 1640 medium supplemented with 5% heat‐inactivated fetal calf serum (Gibco), LPS (0.1 ng/ml), 2 mM L‐glutamine, 1 mM sodium pyruvate and 40 μg/ml gentamycin (Flow laboratories). For antiproliferative test, cells were cultured during 3 days at 2 × 10^5^ cells/ml with different concentrations of extracts (5–20 mg/ml) after which cells were collected, washed and living cells were counted in a cell Coulter Counter ZM equipped with a Coultronic 256 channelizer (Coulter/Beckman). Only cells with diameters ranging from 7 to 14 μm were considered. Cells with diameters inferior or equal to 7 μm (reflecting cell shrinkage) were considered as in necrotic aspect and their numbers were measured by cell coulter counter.

### 2.3 Apoptosis and cell death

Apoptosis was determined by the binding of Annexin V‐Fluorescein isothiocyanate (Annexin V‐FITC, Coulter/Beckman) to phosphatidylserine (PS). In the normal cell, the PS is exposed inside the cell membrane. During apoptosis, this molecule changes position and becomes extracellular. PS is then detected at the surface of cells after 3 days of treatment at 2 × 10^5^ cells/ml. Moreover, propidium iodide (PI) was used to evaluate the necrotic cells according to the manufacturer's instructions. The ratio between Annexin V‐FITC and PI tests revealed the proportion between apoptosis and necrosis aspects.

### 2.4 Cell differentiation to macrophage

In this study, the maturation of cells to the different types of differentiation was verified by the observation of the cell morphology by the staining cyto‐centrifuged cells using a Shandon 3 Cytospin (Thermo Electron) with the Hemacolor kit from Merck and light microscope examination. Additionally, characteristic of cell maturation into macrophages was assessed by the analysis of different related markers like CD11b using the flow cytometer analyzer (Coulter‐Beckman). For that, cells (during 3 days at 2 × 10^5^ cells/ml) were immunostained with an appropriate antibody (Abs) against CD11b marker and the percentages of positive cells and antigen relative density per cell was measured (Merhi et al., 2008). The percentage was obtained by subtracting the peak channel number of the negative control from the peak channel number of the corresponding experimental sample.

## 3 RESULTS AND DISCUSSION

### 3.1 Antiproliferative activity of *A. roseum* extracts U937 cell lines

**FIGURE 1:**
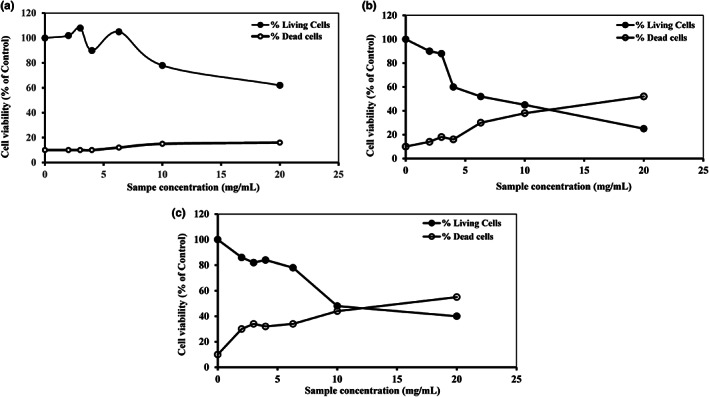
Effects of *A. roseum* extracts on U937 cell proliferation. Cells were cultured during 3 days at 2 × 10^5^ cells/ml with different concentrations of dried (a), crushed (b) and fresh (c) leave extracts (5–20 mg/ml). After incubation, living and dead (necrotic) cells were counted with coulter counter. Results are expressed as percentage (%) of control. Each value represents the mean ± standard deviation of 2–5 independent determinations

Acute myeloid leukemia is one of the major diseases in the world. In that condition, cells present a high capacity to proliferate with a low sensitive aspect to undergoing apoptosis and differentiation. The discovery of various bioactive molecules from plants encourages the use of vegetables in the phytotherapy of cancer. It is well known that processing and preparation conditions can adversely affect the quality of *Allium* extract (Amagase, 2006). For these purposes, we analyzed the antiproliferative potential of aqueous extracts of *A. roseum* from fresh, crushed, and dried leaves (FAE, CAE, and DAE, respectively) on the U937 cell line and its underlying mechanisms. Cells were cultured 3 days in the absence or in the presence of different concentrations of each fraction (5–20 mg/ml). To better estimate the cytotoxic effect of *A. roseum* extracts, the number of necrotic cells with diameters inferior or equal to 7 μm (reflecting cell shrinkage) was measured by Cell Coulter. Our in vitro studies demonstrated that the three studied extracts inhibited cell proliferation in human AML cell line (U937) in a dose‐dependent manner (Figure 1) but FAE and CAE only induced a marked cytotoxic activity and not the DAE. The highest antiproliferative effect is registered at the concentration of 20 mg/ml for FAE and CAE (60% and 73%, respectively). The antiproliferative effect seems to be related to the concentration as well as to the cell line. This was observed in another study where DAE extract showed an important antiproliferative effect against K562 cells (87% of inhibition) using a low concentration equal to 0.5 mg/mL (Souid et al., 2016). The antiproliferative activity against two human colonic adenocarcinoma cell lines (HT29 and CaCo‐2) of the extract of *A. roseum* was studied by Touihri et al. (2016). This study provided interesting antiproliferative activity against HT29 with IC_50_ = 4.64 μg/ml and CaCo‐2 with IC_50_ = 8.22 μg/ml. They mentioned that this potential antiproliferative effect appears to be related to the presence of organosulfur compounds. Despite these interesting premises, most members of the genus *Allium* (especially wild *Allium* species) have not yet been extensively studied for chemical composition and biological activities (Rocchetti et al., 2022).

In addition, several plant‐derived natural products with significant anticancer activity against a wide array of tumor types have been recently isolated, identified, and characterized including, among others, noscapine, bruceantin, and silvestrol (Tawil et al., 2015). One of the hematological malignancies for which novel treatments are urgently needed, and in which the investigation of plant‐derived compounds has been relatively limited thus far is acute myeloid leukemia (AML). Importantly, a number of plant‐derived compounds and extracts have been shown to be active against AML cells, such as *Piper cernuum, Basella alba*, and *H. scoparia* extracts against leukemic cell lines (U937, HL‐60) (Diab et al., 2015; Fraternale et al., 2013; Nguir et al., 2015). In addition, U937 human leukemia cells were also treated with specific bioactive compounds (phenolic vitexin) that induced apoptosis in these cells via a mitochondrial signaling pathway (Lee et al., 2012).The plausible explanation for the fact that different fractions from one plant can be endowed with different antiproliferative activity toward one type of cancer cells is that it has substantially different chemical compositions. For example, *Allium* is known for the presence of the S‐alk(en)yl‐cysteine sulfoxides (RCSO) which has an important antitumor activity (De Gianni & Fimognari, 2015). In this context, antiproliferative activity seems to be related to the change in the level of some compounds like RCSO and to the different processing as compared with other methods of sample preparations as storage conditions of extracts likes time and temperature (Souid et al., 2016). The difference between the anticancer activity of FAE, CAE, and DAE can be explained by the different phytochemical profile (polyphenolic compounds, organosulfur compounds or their precursors…) generated upon each processing of this species.

For example, intact garlic bulbs contain S‐amino acids, including cysteine and methionine (traces), as well as γ‐glutamyl peptides and the alk(en)yl‐cysteine sulfoxides (ACSOs). Thiosulfinates (TSs) (such as Allicin), are formed upon enzymatic hydrolysis of the ACSOs, when raw garlic is cut or crushed. TSs are reactive molecules that can undergo a number of transformations depending on temperature, pH, and solvent of the medium. These reactions can lead to different OSCs, including diallyl, methyl, allyl, and diethyl mono‐, di‐, tri‐, tetra‐, penta‐, and hexasulfides, vinyldithiins, and (E)‐ and (Z)‐ajoene. Allicin (diallyl thiosulfinate/All2TS) is rapidly decomposed to diallyl disulfide (DADS), diallyl sulfide (DAS), diallyl trisulfide (DATS), and sulfur dioxide, and therefore does not seem to be a genuine active compound of garlic. DADS and DATS exert antiproliferative and proapoptotic effects in human epithelial cancer and neuronal cell lines. DADS induces apoptosis in the leukemic HL‐60 cell line through activation of caspase‐3, and inhibits NO synthesis in LPS‐activated macrophages. DATS also stimulates apoptosis of HL‐60 cells and inhibits platelet function by inhibiting platelet aggregation and Ca(2+) mobilization. Other thiosulfinates such as dipropyl thiosulfinate (Pr2TS) and dimethyl thiosulfinate (Me2TS) are mainly identified in onion and leek. Pr2TS, Me2TS, and All2TS are found to inhibit platelet aggregation through inhibition of calpains, by reaction with surface‐free sulfhydryls and internal thiol‐containing proteins (Merhi et al., 2008).

In addition to TSs biosynthesis via the ACSOs‐alliinase interaction, γ‐glutamyl peptides are converted to S‐allylcysteine (SAC) through a different pathway (Merhi et al., 2008).

**FIGURE 2:**
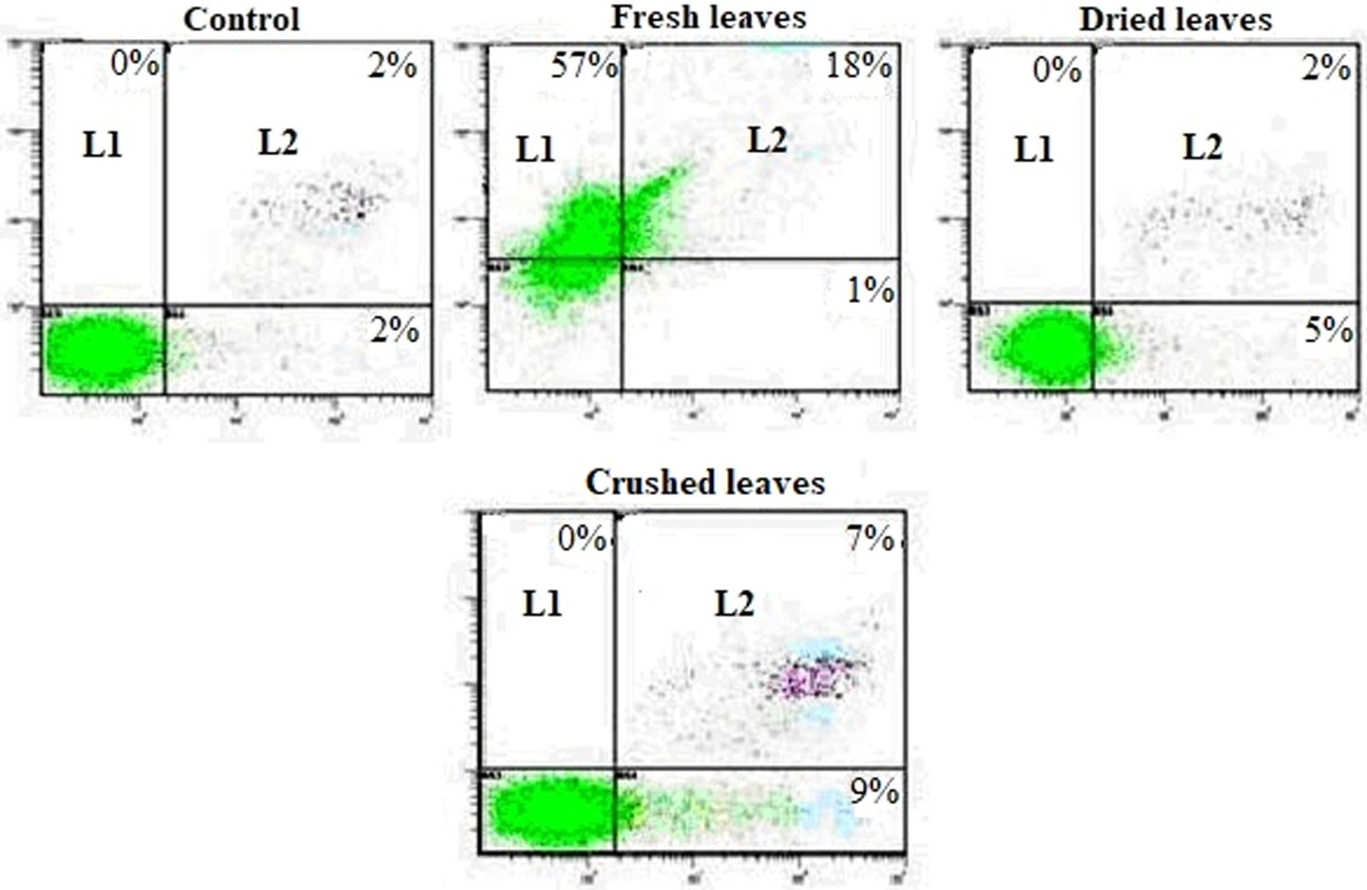
Representative histograms showing flow cytometry analysis of annexin V‐FITC/PI stained with U937 cells. Cells were cultured in the absence (control) or presence of 1/10 FEA, CAE, and DAE on U937 cells (during 3 days at 2 × 105 cells/ml). Results are expressed as log PI fluorescence intensity (y‐axis) vs. log annexin V fluorescence intensity (x‐axis): L1, necrotic cells; L2, secondary necrotic cells; L3, healthy cells; L4, apoptotic cells

Rocchetti et al. (2022) describes the main organosulfur compounds (OSCs) present in whole raw, crushed raw, cooked, distilled, aged, and macerated garlic. Garlic preparations vary substantially in their OSCs composition, raw, or fresh garlic rich in ACSOs, SAC, vinyldithiins, and (E)‐ and (Z)‐ajoene. Crushed garlic is rich in SAC, TS, and vinyldithiins and (E)‐ and (Z)‐ajoene but dried garlic contains all these molecules. It was next investigated whether decreased growth was related to apoptosis. To this end, we analyzed the surface binding of Annexin V, known as an early marker of apoptosis (Arur et al., 2003) with simultaneous PI staining for necrotic cells. Figure 2 shows a representative experiment in which U937 cells were cultured for 3 days in the absence or presence of FAE, CAE, and DAE (20 mg/mL). U937 cells treated with FAE or DAE or CAE were found weakly positive for Annexin V (L4: 1%, 5%, and 9%, respectively), and for both Annexin V and PI (L2: 18%, 2%, and 7%, respectively). As for the antiproliferative effect, the capacity of extract to produce apoptosis and/or necrosis seems to be related to the concentration as well as to cell line. In fact, even at 300, 600, and 900 μg/ml K562 was sensitive to extract and showed an important percentage of Annexin‐positive cells equal to 27.4, 32.3, and 38%, respectively compared with the non‐treated ones (Souid et al., 2016). Moreover, the present study showed that on the one hand, apoptotic cells were more present with CAE and on the other hand necrotic cells were more observed with FAE.

### 3.2 Analysis of differentiation markers

**FIGURE 3:**
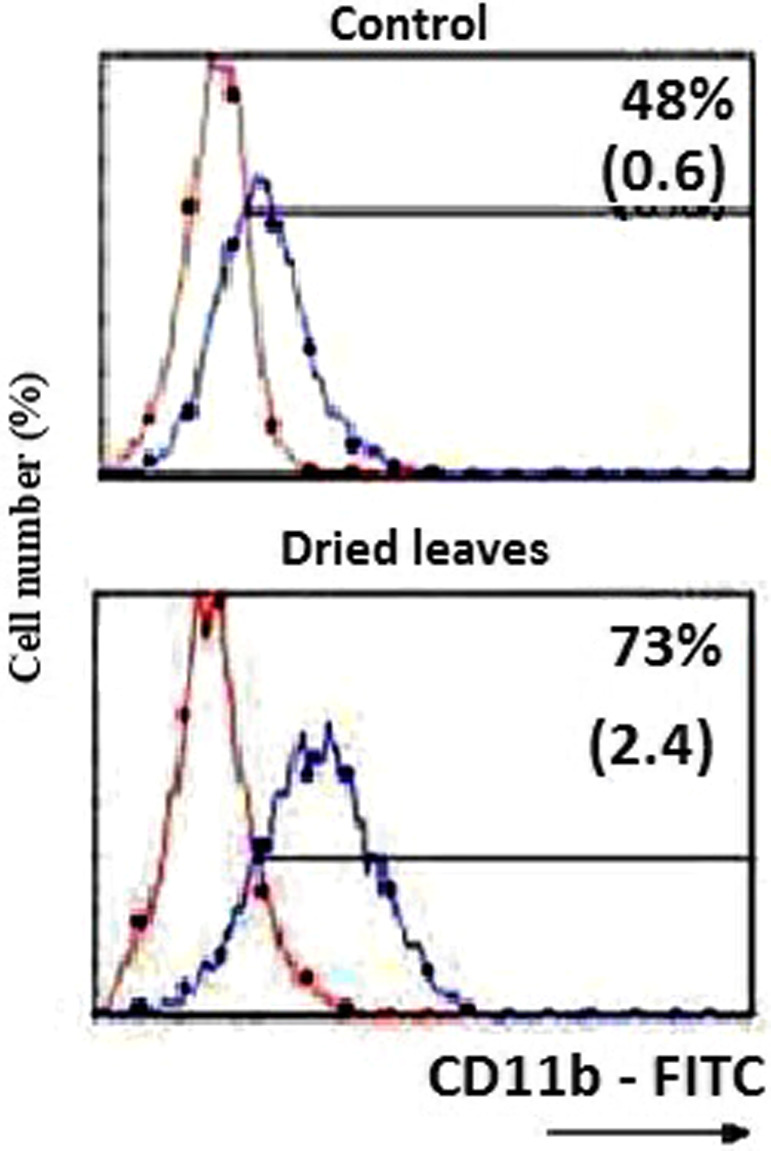
Percentages of immunostained U937cells with an appropriate antibody (abs) against CD11b marker using the extract DAE. Cells were cultured during 3 days at 2 × 10^5^ cells/ml in the absence (control) or in the presence of *a*. roseum extracts (5 μM) (1 ng/ml)

It is widely recognized that apoptosis is initiated by two principal pathways: a mitochondrion‐mediated intrinsic pathway and a death‐receptor‐induced extrinsic pathway (Al Sinani & Eltayeb, 2017). In this study, the capacity of extract to enhance the expression of CD11b antigen in treated U937 cells was investigated (Figure 3). By microscopic observation, results showed a change in morphology (increase in cell size, decrease in the nuclear/cytoplasmic ratio and apparition of vacuolization) consistent with macrophage differentiation. Moreover, immune fluorescence in a Coulter flow cytometer permitted us to view that DAE is responsible of a high production of CD11b antigen (73% of cells with CD11b marker as a function of control). This is confirming that *A. roseum* leaves extract is associated with a monocyte/macrophage differentiation. At day 3, the block in growth of only DAE‐treated U937 cells was correlated with a marked enhanced expression of CD11b as compared with untreated cells (Figure 3). These findings are in line with results obtained by other groups testing dipropyl TS and dimethyl TS, two sulfur‐containing molecules thiosulfinates in AML, shown to induce a marked shift toward a macrophage morphology associated with the enhanced expression of CD11b in the U937 AML cell lines (Merhi et al., 2008). This result confirms that the activity of the extract was probably due to the presence of Pr2 TS and Me2 TS in the DAE extract. Specific fluorescence intensity of CD11b antigen (black line) was detected by immune fluorescence in a Coulter flow cytometer as described in Materials and methods section. Staining of cells with a matched isotype antibody (mIgG1) served as the respective negative control (gray area). Results are expressed as relative cell number (y‐axis) vs. log fluorescence intensity (x‐axis). Values represent percentage of positive cells. One experiment representative of four different experiments is shown.

## 4 CONCLUSION

The development of new drugs in phytomedicine based on functional food ingredients and/or food supplements is an effective alternative to conventional chemotherapy and *A. roseum* could be a promising source of natural products for industrial applications such as production of pharmaceuticals.

## CONFLICT OF INTEREST

No potential conflict of interest was reported by the authors.

## References

[fsn32965-bib-0001] Al Sinani, S. S. S. , & Eltayeb, E. A. (2017). The steroidal glycoalkaloids solamargine and solasonine in solanum plants. South African Journal of Botany, 112, 253–269. 10.1016/j.sajb.2017.06.002

[fsn32965-bib-0002] Amagase, H. (2006). Clarifying the real bioactive constituents of garlic. The Journal of Nutrition, 136(3), 716S–725S. 10.1093/jn/136.3.716S 16484550

[fsn32965-bib-0003] Arur, S. , Uche, U. E. , Rezaul, K. , Fong, M. , Scranton, V. , Cowan, A. E. , Mohler, W. , & Han, D. K. (2003). Annexin I is an endogenous ligand that mediates apoptotic cell engulfment. Developmental Cell, 4, 587–598. 10.1016/s153-5807(03)00090-x 12689596

[fsn32965-bib-0004] Boubakri, A. , Leri, M. , Bucciantini, M. , Najjaa, H. , Ben Arfa, A. , Stefani, M. , & Neffati, M. (2020). *Allium roseum* L. extract inhibits amyloid beta aggregation and toxicity involved in Alzheimer's disease. PLoS One, 15(9), e0223815. 10.1371/journal.pone.0223815 32997672 PMC7526880

[fsn32965-bib-0005] De Gianni, E. , & Fimognari, C. (2015). Chapter seven ‐ anticancer mechanism of sulfur‐containing compounds. The Enzymes, 37, 167–192. 10.1016/bs.enz.2015.05.003 26298460

[fsn32965-bib-0006] Diab, K. A. , Shafik, R. E. , & Yasuda, S. (2015). *In‐vitro* antioxidant and antiproliferative activities of novel orange peel extract and it's fractions on leukemia HL‐60 cells. Asian Pacific Journal of Cancer Prevention, 16(16), 7053–7060. 10.7314/apjcp.2015.16.16.7053 26514490

[fsn32965-bib-0007] Fraternale, D. , Ricci, D. , Calcabrini, C. , Guescini, M. , Martinelli, C. , & Sestili, P. (2013). Cytotoxic activity of essential oils of aerial parts and ripe fruits of *Echinophora spinosa* (Apiaceae). Natural Product Communication, 8, 1645–1649. 10.1177/1934578X1300801137 24427963

[fsn32965-bib-0008] Hassanpour, S. H. , & Karami, S. Z. (2018). The role of plant extracts in the treatment of leukemia types. International Journal of Pharmacognosy, 5(2), 74–81. 10.13040/IJPSR.0975-8232

[fsn32965-bib-0009] Hosono, T. , Fukao, T. , Ogihara, J. , Ito, Y. , Shiba, H. , Seki, T. , & Ariga, T. (2005). Diallyl trisulfide suppresses the proliferation and induces apoptosis of human colon cancer cells through oxidative modification of beta‐tubulin. Journal of Biological Chemistry, 280(50), 41487–41493. 10.1074/jbc.M507127200 16219763

[fsn32965-bib-0010] Hsiao, E. Y. , McBride, S. W. , Hsien, S. , Sharon, G. , Hyde, E. R. , McCue, T. , Codelli, J. A. , Chow, J. , Reisman, S. E. , Petrosino, J. F. , Patterson, P. H. , & Mazmanian, S. K. (2013). Microbiota modulate behavioral and physiological abnormalities associated with neurodevelopmental disorders. Cell, 155(7), 1451–1463. 10.1016/j.cell.2013.11.024 24315484 PMC3897394

[fsn32965-bib-0011] Katrin, K. , Wobbeke, W. , Frauke, N. , Andrea, H. , Christoph, V. T. , & Hans, D. (2017). The health effects of aluminum exposure. Deutsches Ärzteblatt International, 114(39), 653–659. 10.3238/arztebl.2017.0653 29034866 PMC5651828

[fsn32965-bib-0013] Lanzotti, V. (2006). The analysis of onion and garlic. Journal of Chromatography A, 1112, 3–22. 10.1016/j.chroma.2005.12.016 16388813

[fsn32965-bib-0014] Lee, S. H. , Yang, J. , Goddard, M. E. , Visscher, P. M. , & Wray, N. R. (2012). Wray estimation of pleiotropy between complex diseases using single‐nucleotide polymorphism‐derived genomic relationships and restricted maximum likelihood. Bioinformatics, 28(19), 2540–2542. 10.1093/bioinformatics/bts474 22843982 PMC3463125

[fsn32965-bib-0015] Merhi, F. , Auger, J. , Rendu, F. , & Bauvois, B. (2008). *Allium* compounds, dipropyl and dimethylthiosulfinates as antiproliferative and differentiating agents of human acute myeloid leukemia cell lines. Biologics: Targets & Therapy, 2(4), 885–895. 10.2147/btt.s3212 19707466 PMC2727902

[fsn32965-bib-0016] Milner, J. A. (2006). Preclinical perspectives on garlic and cancer. The Journal of Nutrition, 136(3), 827S–831S. 10.1093/jn/136.3.727S 16484574

[fsn32965-bib-0017] Najjaa, H. , Neffati, M. , Zouari, S. , & Ammar, E. (2007). Essential oil composition and antibacterial activity of different extracts of *Allium roseum* L. a north African endemic species. Comptes Rendus Chimie, 10(9), 820–826. 10.1016/j.crci.2007.03.003

[fsn32965-bib-0018] Najjaa, H. , Zouari, S. , Ammar, E. , & Neffati, M. (2011). Phytochemical screening and antibacterial properties of *Allium roseum* L. a wild edible species in North Africa. Journal of Food Biochemistry, 35, 699–714. 10.1111/j.1745-4514.2010.00411.x

[fsn32965-bib-0019] Najjaa, H. , Zria, K. , Fattouch, S. , Ammar, E. , & Neffati, M. (2011). Antioxidant and antimicrobial activities of *Allium roseum* L. “Lazoul” a wild edible endemic species in North Africa. International Journal of Food Properties, 14(2), 371–380. 10.1080/10942910903203164

[fsn32965-bib-0020] Nguir, A. , Znati, M. , Garrab, M. , Flamini, G. , Hamza, M. A. , & Ben Jannet, H. (2015). Hydrodistillation kinetic and biological investigations of essential oils from the Tunisian *Crithmum maritimum* L. Journal of the Tunisan Chemical Society, 17, 83–94.

[fsn32965-bib-0021] Rocchetti, G. , Zhang, L. , Bocchi, S. , Giuberti, G. , Ak, G. , Elbasan, F. , Yıldıztugay, E. , Ceylan, R. , Picot‐Allain, M. C. N. , Mahomoodally, M. F. , Lucini, L. , & Zengin, G. (2022). The functional potential of nine allium species related to their untargeted phytochemical characterization, antioxidant capacity and enzyme inhibitory ability. Food Chemistry, 368, 130782. 10.1016/j.foodchem.2021.130782 34392121

[fsn32965-bib-0022] Siegel, R. L. , Fedewa, S. A. , Miller, K. D. , Goding‐Sauer, A. , Pinheiro, P. S. , Martinez‐Tyson, D. , & Jemal, A. (2015). Cancer statistics for Hispanics/Latinos. CA: A Cancer Journal for Clinicians, 65(6), 457–480. 10.3322/caac.21314 26375877

[fsn32965-bib-0023] Souid, S. , Najjaa, H. , Riahi‐Chebbi, I. , Haoues, M. , Neffati, M. , Arnault, I. , Auger, J. , Karoui, H. , Essafi, M. , & Essafi‐Benkhadir, K. (2016). *Allium roseum* L. extract exerts potent suppressive activities on chronic myeloid leukemia K562 cell viability through the inhibition of BCR‐ABL, PI3K/Akt and ERK1/2 pathways and the abrogation of VEGF secretion. Nutrition and Cancer: An International Journal, 69(1), 1–14. 10.1080/01635581.2017.1248295 27892697

[fsn32965-bib-0024] Tawil, M. , Bekdash, A. , Mroueh, M. , Daher, C. F. , & Abi‐Habib, R. J. (2015). Wild carrot oil extract is selectively cytotoxic to human acute myeloid leukemia cells. Asian Pacific Journal of Cancer Prevention, 16(2), 761–767. 10.7314/apjcp.2015.16.2.761 25684522

[fsn32965-bib-0025] Touihri, I. , Boukhris, M. , Marrakchi, N. , Luis, J. , Hanchi, B. , & Kallech‐Ziri, O. (2016). Chemical composition and biological activities of *allium roseum* L. var. *grandiflorum Briq*. Essential oil. Journal of Oleo Science, 64(8), 869–879. 10.5650/jos.ess15055 26179004

[fsn32965-bib-0026] Xiao, D. , & Singh, S. V. (2006). Diallyl trisulfide, a constituent of processed garlic, inactivates Akt to trigger mitochondrial translocation of BAD and caspase‐mediated apoptosis in human prostate cancer cells. Carcinogenesis, 27(3), 533–540. 10.1093/carcin/bgi228 16169930

[fsn32965-bib-0027] Xiao, D. , Pinto, J. T. , Gundersen, G. G. , & Weinstein, I. B. (2005). Effects of a series of organosulfur compounds on mitotic arrest and induction of apoptosis in colon cancer cells. Molecular Cancer Therapeutics, 4(9), 1388–1398. 10.1158/1535-7163.MCT-05-0152 16170031

[fsn32965-bib-0028] Zhang, Q. L. , Jia, L. , Jiao, X. , Guo, W. L. , Ji, J. W. , Yang, H. L. , & Niu, Q. (2012). APP/PS1 transgenic mice treated with aluminum: An update of Alzheimer's disease model. International Journal of Immunopathology and Pharmacology, 25, 49–58. 10.1177/039463201202500107 22507317

